# A Method for Predicting Protein-Protein Interaction Types

**DOI:** 10.1371/journal.pone.0090904

**Published:** 2014-03-13

**Authors:** Yael Silberberg, Martin Kupiec, Roded Sharan

**Affiliations:** 1 Department of Molecular Microbiology and Biotechnology, Tel-Aviv University, Tel Aviv, Israel; 2 The Blavatnik School of Computer Science, Tel-Aviv University, Tel-Aviv, Israel; Huazhong University of Science and Technology, China

## Abstract

Protein-protein interactions (PPIs) govern basic cellular processes through signal transduction and complex formation. The diversity of those processes gives rise to a remarkable diversity of interactions types, ranging from transient phosphorylation interactions to stable covalent bonding. Despite our increasing knowledge on PPIs in humans and other species, their types remain relatively unexplored and few annotations of types exist in public databases. Here, we propose the first method for systematic prediction of PPI type based solely on the techniques by which the interaction was detected. We show that different detection methods are better suited for detecting specific types. We apply our method to ten interaction types on a large scale human PPI dataset. We evaluate the performance of the method using both internal cross validation and external data sources. In cross validation, we obtain an area under receiver operating characteristic (ROC) curve ranging from 0.65 to 0.97 with an average of 0.84 across the predicted types. Comparing the predicted interaction types to external data sources, we obtained significant agreements for phosphorylation and ubiquitination interactions, with hypergeometric p-value = 2.3e^−54^ and 5.6e^−28^ respectively. We examine the biological relevance of our predictions using known signaling pathways and chart the abundance of interaction types in cell processes. Finally, we investigate the cross-relations between different interaction types within the network and characterize the discovered patterns, or motifs. We expect the resulting annotated network to facilitate the reconstruction of process-specific subnetworks and assist in predicting protein function or interaction.

## Introduction

Protein-protein interactions (PPIs) play a key role in a diverse range of biological processes. As proteins interact in a specific manner, a large number of interaction types exists [1]. Understanding the nature and mechanism of these interactions is crucial in deciphering cellular processes at the molecular level. To explore the molecular mechanisms of protein interactions and chart a cell-wide interaction map, many experimental techniques have been developed to date. The different methods make use of biochemical, biophysical and imaging techniques in order to monitor interactions in scales ranging from single proteins (e.g., confocal microscopy) to genome wide screening (e.g., yeast two-hybrid and tandem affinity purification). Presently, more than half a million interactions are cataloged in public databases [2], calling for computational methods to categorize them according to their different types.

Several previous attempts were made to classify protein interactions into different types. One approach exploits protein structural information in order to predict protein docking mode and subsequently the interaction type [3]–[5]. Another approach makes use of sequence data of the interacting interface in order to predict interaction types [6], [7]. For example, [7] explores the difference in amino acid compositions and residue-residue preferences of protein interfaces to classify protein interactions into six types: homo- and hetero- structural domain, obligate and transient interaction and homo- and hetero- oligomers. A caveat of these approaches is that they rely on prior structural information; in addition they use a coarse classification to high level classes of types.

Here we propose a method for predicting interaction type based on the experimental techniques by which the interaction was detected, using no prior biological information. We compiled a dataset of 180,353 PPIs and their corresponding detection methods from multiple databases and applied logistic regression to predict for each PPI its interaction types. We applied this method separately on ten interaction types including covalent binding, disulfide bond, protein cleavage and cleavage, deacetylation, dephosphorylation, methylation, phosphorylation, ubiquitination and ADP ribosylation. We validated our predictions using internal cross-validation and external data sources, obtaining high areas under the ROC curve. In addition, we chart the distribution of interaction types in biological pathways, showing that different cellular processes tend to be mediated by different interaction types. Finally, we illuminate inter-relations between types by analyzing recurrent network motifs spanning multiple types.

## Results

**Figure 1:**
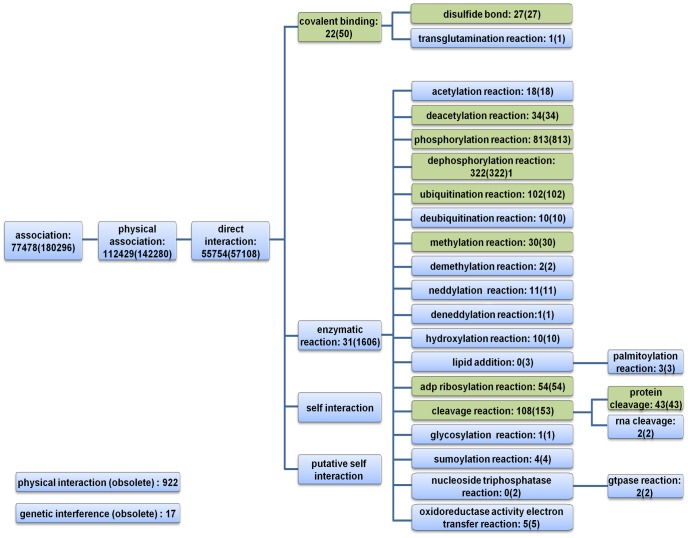
Hierarchical view of psi-mi interaction types extracted from PPIs records. For every interaction type we state the number of PPIs directly associated to the type. The number of PPIs associated with the type and its descendants in the ontology is given in parenthesis. Interaction types predicted using logistic regression are highlight in green.

We compiled a human protein-protein interactions dataset from multiple sources utilizing the psi-mi format [8]. Interaction types and interaction detection methods were extracted for each PPI. The psi-mi format provides a hierarchical representation of the types, where at the highest level there are four categories: (i) association, (ii) colocalization, (iii) genetic interaction and (iv) predicted interaction. Querying for interactions of the type *‘association’* and its descendants we obtained 461,742 redundant interactions corresponding to 180,353 unique PPIs (see Methods). Each PPI record was associated with up to 20 detection methods and 5 interaction types with an average of 1.4 and 1.38 associations, respectively. When accounting for ancestors in the hierarchical structure, the average PPI-detection method and interaction type associations increased to 6.8 and 3.1 respectively. Figure 1 summarizes the interaction types extracted from the PPI records.

We hypothesized that different detection methods are better suited for different types. To test this hypothesis we examined the enrichment of interaction types detected by the different methods. We found that ‘affinity chromatography’, was underrepresented with enzymatic reaction and several of its descendant categories (hypergeometric 1-p-value = 1.3e^−18^, 1.2e^−17^,1.1e^−07^,8.1e^−07^,1.5e^−08^ for enzymatic reaction, cleavage reaction, protein cleavage, dephosphorylation reaction and ADP ribosylation reaction, respectively). On the other hand, covalent binding and disulfide bond were both enriched with this method (hypergeometric p-value = 1.3e^−18^and 2.6e^−11^ respectively), suggesting that this detection method is less suitable for detecting transient enzymatic reactions, and is better suited for the detection of protein complexes. Notably, ‘ubiquitination reaction’ was also found to be enriched with affinity chromatography (p-value = 2.1e^−9^), reflecting the need for stable interactions to carry out ubiquitination reactions. Similarly, ‘two hybrid array’ was found to be underrepresented in phosphorylation and dephosphorylation interactions (p-value = 2.3e^−5^ and 2.5e^−7^, respectively), while enriched in ubiquitination reactions (p-value = 2e^−24^), demonstrating again the subtle differences between these types of enzymatic reactions. Finally, ‘Comigration in gel electrophoresis’ showed enrichment in identifying covalent binding interactions (p-value = 1.1e^−16^) while being underrepresented in enzymatic reactions (p-value = 1.1e^−16^), in accordance with the technique prevalent use [9]. A list of top enrichments and underrepresentations is given in Table S1.

### Predicting Interaction Types

Next, we attempted to predict the interaction type of a PPI from its associated detection methods, focusing on two main branches of the type hierarchy: (i) covalent binding and (ii) enzymatic reaction (see Figure 1). To this end, we trained a generalized linear regression model based on 1,558 PPIs annotated to these types (see Methods).

**Figure 2:**
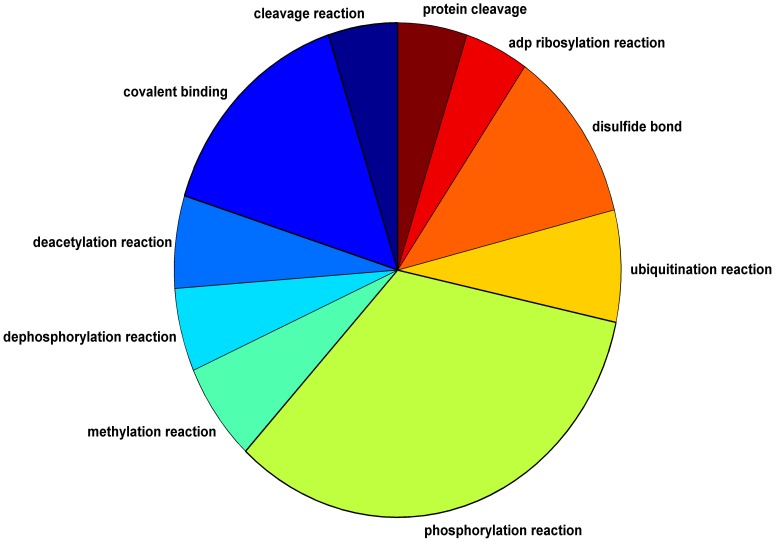
distribution of predicted interaction types.

To evaluate our prediction method, we used a 10-fold cross-validation setting. Our method obtained an area under the receiver-operating-characteristic curve (AUC) of 0.84 on average across the different interaction types (see Table 1). To determine a set of predicted interactions for each type we used a cutoff point which maximizes the sum of specificity and sensitivity. Under this cut-point, we were able to predict at least one interaction type for 108,821 interactions (60%). Figure 2 displays the distribution of predicted interaction types.

**Table 1:** Areas under the receiver-operating-characteristic curves.

Interaction Type	Number of known interaction	Size of negative set	AUC
cleavage reaction	153	153	0.82
covalent binding	50	100	0.86
deacetylation reaction	34	100	0.89
dephosphorylation reaction	322	322	0.71
methylation reaction	30	100	0.81
phosphorylation reaction	813	813	0.65
ubiquitination reaction	102	102	0.87
disulfide bond	27	100	0.84
adp ribosylation reaction	54	100	0.97
protein cleavage	43	100	0.97

Areas under the curves (AUC) obtained in a 10-fold cross-validation setting. The AUC is averaged across 20 cross validation repeats.

We further validated our predictions against two external data-sources storing specific interaction types: (i) phospho.ELM [10], a manually curated database of phosphorylation sites and their kinases; and (ii) hUbiquitome [11], a database of experimentally verified human ubiquitination enzymes and substrates.

To assess our predictions, we compared the set of predicted phosphorylation interactions to a set of 479 gold standard phosphorylation interactions stored in Phospho.ELM (see Methods), obtaining an AUC of 0.70. Choosing a cutoff as above, we were able to retrieve 75% of the gold standard interactions with an accuracy of 58%, yielding a hyper geometric p-value <2.3e^−54^. In a similar manner, comparing the classifier ranks for ubiquitination interactions to a set of 203 gold standard interactions retrieved from hUbiquitome yielded an AUC of 0.69. The set of predicted ubiquitination interactions was highly enriched in gold-standard PPIs, with hyper geometric p-value = 5.6e^−28^, retrieving 37% of gold standard proteins with an accuracy of 91%.

Next, we tested the functional similarity of the proteins participating in the different interaction types. We compared the semantic similarity of proteins mediating each interaction type to the similarity of a randomly selected group of interacting proteins of the same size (see Methods). The similarity score distribution was significantly higher for proteins spanning the same interaction type (Wilcoxon p-value = 0 for all interaction types using GO biological process, cell compartment and molecular function).

To analyze the categories enriched with each interaction type, we focused on the top 100 proteins with the highest network degree in each of the types (see Methods). All interaction types were highly enriched in GO categories, with at least 69 enriched categories per type and a total of 1758 enrichment over all types (FDR <0.05). Reassuringly, we found that most interaction types were enriched with terms directly describing their predicted type. For example, ‘protein cleavage’ were enriched in ‘proteolysis involved in cellular protein catabolic process’; and ‘proteolysis’ categories. Both ‘disulfide bond’ and ‘covalent binding’ proteins were enriched in categories involving large complexes such as ‘ribosome’, ‘ribosomal subunit’, ‘ribonucleoprotein complex’ and more generally in the category ‘macromolecular complex’. ‘Ubiquitination reaction’ was enriched in ‘protein ubiquitination’ and ‘ubiquitin-protein ligase activity’. Proteins involved in phosphorylation reactions were enriched in eight GO categories directly engaging phosphorylation including ‘protein amino acid phosphorylation’, ‘phosphorylation’ and ‘protein kinase activity’.

### Interaction Types in Cellular Processes

To assess the relevance of the predicted interaction types to cellular processes, we download KEGG pathways [12] and evaluated the distribution of interaction types within pathways (see Methods, 89 pathways spanning 10 sub-categories). First, we computed Pearson correlation between the number of interactions of each type across pathways. We found that the correlations of pathways within the same subcategories were significantly higher than the correlations of pathways in different subcategories (0.72 and 0.63 respectively, Wilcoxon p-value = 1.8e^−18^) suggesting that different cell processes are mediated by different interaction types. Repeating the analysis using KEGG-type annotations revealed the same trend with an intra-subcategory correlation of 0.64 vs. inter-subcategory correlation of 0.32 (Wilcoxon test p-value = 4.7e^−48^).

Next, we compared the predicted interaction types to KEGG’s annotations. KEGG interaction types consist of 13 annotations, from which five are molecular events and three were comparable with predicted types (see Methods). We first tested the enrichment of predicted types within the corresponding KEGG types. All three interaction types were enriched with the corresponding KEGG types (hypergeometric p-value = 1.7e^−35^, 0.02 and 1.7e^−16^ for phosphorylation, dephosphorylation and ubiquitination reactions, respectively). Moreover, we found that the abundance of predicted interactions of type phosphorylation, dephosphorylation and ubiquitination across pathways were correlated with their abundance as annotated in KEGG pathways (Pearson correlation = 0.54, 0.67 and 0.19 respectively). Surprisingly, we observed a high correlation between interactions annotated in KEGG as ‘activation’ and all enzymatic reactions (e.g. cleavage, dephosphorylation and methylation, with Pearson correlation = 0.61, 0.61 and 0.52 respectively). Intrigued by this correlation, we wondered if our predicted interaction types can explain the mechanism of action for interactions annotated as ‘activation’/‘inhibition’ by KEGG, many of which are of unknown nature. One example for such an interaction is the inhibition of Notch by Numb in the ‘Notch signaling pathway’. It is known that Numb can bind to the intercellular domain of Notch, but the specific mechanism by which it inhibits Notch is still under investigation [13]. We predict this interaction to be of cleavage type. Indeed, a recent study has shown that the overexpression of Numb reduces intracellular pools of biotinylated Notch1 by promoting Notch1 degradation [14]. Additionally, we predicted the activation interaction of Presenilin- Notch in the same pathway, to be of cleavage type as well. Indeed, evidence supports that presenilin-1 has a proteolytic effect on Notch, causing its activation [15]. Similarly, we predicted that the inhibitory interaction Mdm2 - p53 in the ‘chronic myeloid leukemia’ pathway is a ubiquitination reaction. Indeed, Mdm2 serves as a p53-specific ubiquitin ligase, using this mechanism to inhibit p53 [16]. Finally, the activation interaction between mTOR and RPS6KB1 in the ‘acute myeloid leukemia’ pathway was predicted to be of phosphorylation type by our method. Indeed this interaction is a well characterized phosphorylation interaction [17]. These examples demonstrate that the specific enzymatic reaction predicted using our method can add complementary information to the annotation assigned by KEGG, revealing the specific nature of the interaction.

### Interaction Type Motifs

Network motifs are simple patterns of interaction in networks that occur more frequently than expected. We searched for three-node patterns by exhaustively enumerating all three proteins interconnected by three PPIs as suggested by [18]. We compared the number of patterns found to those formed by 500 randomized PPI networks (see Methods). Eight enriched motifs were found (empirical p-value <0.05). All motifs consisted of at least two interactions of the same type and three of them were a single-type motif (see Table 2).

**Table 2:** Significantly recurring network motifs.

interaction A	interaction B	interaction C
cleavage reaction	cleavage reaction	cleavage reaction
covalent binding	cleavage reaction	cleavage reaction
phosphorylation reaction	cleavage reaction	cleavage reaction
phosphorylation reaction	phosphorylation reaction	cleavage reaction
covalent binding	covalent binding	covalent binding
phosphorylation reaction	covalent binding	covalent binding
methylation reaction	methylation reaction	covalent binding
phosphorylation reaction	phosphorylation reaction	phosphorylation reaction

One example of a single-type motif consists of three covalent bonds between three proteins, representing a protein complex. For instance the mediator complex, a multiprotein complex composed of at least 26 subunits [19], was highly represented in this motif, having 246 covalent-bond triplets of the complex subunits in our predictions.

A second example consists of three cleavage reactions between three proteins. This motif suits, for example, the pattern of caspase activation in the apoptotic process. Caspases, cysteine proteases which play essential roles in apoptosis, are activated by cleavage of their precursors [20]. We found Caspase - 6, 7 and 8 to form a cleavage motif. It is known that initiator caspase 8 triggers the apoptotic pathways by cleaving effector caspases precursors, including caspases 6 and 7 [21]. Additionally, evidence suggests that the effector caspases 6 and 7 have a mutual activation and amplification effect on each other [20]. Moreover, we found caspase 6 and 7 to form a motif with SP1. Caspases 6 and 7 act as executioner proteins of apoptosis, sharing many common cellular targets, whose processing leads to cell degradation. SP1 is a transcription factor involved in many cellular processes including cell growth, differentiation and apoptosis, making it a likely candidate for degradation by apoptotic caspases.

Another class of motifs contains motifs with two interactions of the same type. An example of such a motif consists of two cleavage reactions and a covalently-linked pair. This motif may represent cases in which cleaving is carried out on two members of a complex, or in which two proteins collaborate in carrying out a cleavage reaction. We found for example, that the DEAD box protein 39, a protein involved in RNA processing [22], forms ‘double cleavage-covalent binding’ motifs with ribosomal proteins (e.g., 60S ribosomal protein L7, L4, L12 and more). Indeed, DEAD box proteins are known to be involved in ribosome biogenesis [23].

A second example consists of a kinase which phosphorylates two proteins interconnected by a cleavage reaction. This motif implies that phosphorylation and cleavage reactions may operate in a coordinated manner in signaling pathways as previously suggested [24]–[26].

## Conclusions

In this work, we presented a new method for predicting protein interaction type. Our method exploits the experimental techniques by which interactions were detected to infer their functional type. We first demonstrated how experimental techniques are differently correlated with interaction types. Next, we examined the distribution of interaction types in known pathways, showing that different cell processes are mediated via different interaction type compositions. Finally, we utilized the resulting type-annotated network to elucidate interaction type motifs. The motifs uncovered simple patterns of common processes in the cell mediated by different types of protein interactions and provide a glimpse into the interactome’s underlying structure. All motifs consist of at least two interactions of the same type, indicating that interactions of the same type tend to form functional groups in the cell.

Our approach exploits the delicate preferences of a experimental approach in detecting different interaction types to systematically predict interaction types. While some detection methods may be biased toward specific interaction types, the vast majority of interactions in the dataset are taken from large scale exploratory experiments which are not a priori biased toward a given set of interactions. Specifically, 99% of the interactions in the dataset were detected in large scale experiments reporting over 1,000 PPIs (58% of which were also detected in small scale experiments). This suggests that our method relies on unbiased experiments and its strength might stem from the integration of multiple detection methods to tackle the prediction task.

To the best of our knowledge, our method is the first to predict interaction functional type. We expect that as more interaction types will become predictable, the interaction profile of a protein could illuminate protein function as well as the way it mediates its role within the cell.

We note that additional attributes can be exploited to improve interaction type prediction. For example, properties such as protein’s functional annotations can be used in the learning process, assuming that proteins with similar properties tend to interact in a similar manner.

## Materials and Methods

### Data Assembly

Protein-protein interactions (PPIs) were downloaded from multiple sources using the molecular interactions query language (MIQL 2.5) of PSICQUIC [27]. PSICQUIC enables programmatic access to molecular interaction databases supporting the psi-mi format, which provides a hierarchical structure describing protein interactions. The resulting PPI compendium spans 461,742 physical PPIs in humans, representing 180,353 unique interactions for 19,584 proteins. In total, 130 psi-mi interaction detection methods and 29 interaction types were extracted from the PPI compendium, from which only 0.8% of the interactions (1,492 interactions) were classified to specific interaction types (i.e. “enzymatic reaction” and “covalent binding” and their descendants), while the rest were classified to high level interaction type (e.g. “*physical association*”), see Figure 1. Additionally, we manually mapped interactions detected using psi-mi 0415 “*enzymatic study*” descendant terms (e.g. phosphatase assay, cleavage assay, etc.) which are designed to detect specific interaction types, to the corresponding interaction type, resulted in 1,656 unique interactions with known interaction type.

In order to compile a complete detection method vector we exploited the hierarchical structure of the psi-mi format, to associate PPI with ancestors of the detection method to which it was annotated.

In a similar manner PPIs were associated with interaction types ancestors, resulting in 27 redundant predictions of ‘disulfide bond’ and ‘covalent bond’ and 43 redundant predictions of ‘protein cleavage’ and ‘cleavage reaction’ (see Figure 1).

### Training Logistic Regression

We trained a generalized linear regression model, using a MATLAB classifier with binomial distribution. To avoid trivial prediction cases all descendants of interaction detection method psi-mi 0415 “*enzymatic study*” (e.g. phosphatase assay, cleavage assay, etc.) which are designed to detect specific interaction types, were dismissed from the detection method vector. Additionally, rare detection methods associated with less than 200 interactions and high-level detection methods associated with all interactions (i.e. *‘experimental interaction detection’* and *‘interaction detection method’*) were dismissed from the analysis, resulting in a vector of 39 detection methods serving as feature vector to the logistic regression (see Table S2). For the prediction task we focused on all types representing specific interactions types, i.e., *‘enzymatic reaction’*, *‘covalent binding’* and their descendants dismissing high level interaction of type ‘direct interaction’ and its ancestors. Additionally, interaction types assigned to less than 20 PPIs were ignored. Last, we dismissed *‘Enzymatic reaction’* from the prediction analysis as 1,606 (97%) of the known interaction type were annotated to this type making its prediction redundant and causing the lack of an appropriate negative set.

The filtering process resulted in a set of 1,558 unique protein interactions with predictable type. The positive-training set for each interaction type included all interactions associated with that interaction type, while the negative set included interactions associated with different specific interaction types. Additionally, as interactions annotated to a specific type represent less than 1% of the interaction dataset, we accounted for random interactions not associated with any specific type (i.e., interaction annotated only to *‘physical association’* or *‘direct interaction’*) as a part of the negative set. To gain a wider variety of interaction types and detection methods we chose the random interactions from the set of interaction associated with at least four detection methods. These 139,961 interactions (78% of the complete dataset) forms a representative group of reliable and well annotated interactions. The size of the negative set was set to the size of the positive set or at least 100 interactions, see Table 1.

### Evaluating Predictions

We preformed 20 independent 10-fold cross-validation schemes, choosing in each of them different random negative sets and partitioning the data into different random groups.

To evaluate the predicted phosphorylation interactions we downloaded human kinase-substrate interaction from Phospho.ELM. We were able to uniquely map 546 interactions stored in Phospho.ELM to interaction in our data set, from which 67 interactions were psi-mi annotated to *‘phosphorylation reaction’* and were therefore removed from the analysis.

In a similar manner we downloaded 413 interactions from hUbiquitome, 208 of which were uniquely mapped to interactions stored in our data set, from which 5 where annotated as *‘ubiquitination reaction’* and where therefore removed from the gold standard set.

### Validation using GO

For each interaction type, we calculated Resnik’s similarity among proteins spanning the interaction type subnetwork. The random set was composed of protein interactions randomly chosen from the complete PPI network. The random PPIs set was of the same size of the predicted PPIs set for that type. We repeated the procedure with ten random groups for each interaction type, reporting the average similarity of the ten reported groups with maximal Wilcoxon p-value obtained over all iterations.

Enrichment analysis was done using DAVID [28]. In each interaction type, the top hundred proteins having the largest number of interactions were chosen, and compared to a set of 19,584 proteins spanning the complete PPI network. The enrichment was performed on all GO ontology categories.

### Comparison with KEGG

KEGG pathways in humans were retrieved using KEGG API [29]. There are 13 protein interaction relations annotated in KEGG xml, from which five enzymatic reactions, including: ‘compound’, ‘activation’, ‘inhibition’, ‘indirect effect’, ‘state change’, ‘binding/association’, ‘dissociation’, ‘missing interaction’ and enzymatic reactions: ‘phosphorylation’, ‘dephosphorylation’, ‘glycosylation’, ‘ubiquitination’ and ‘methylation’. The last five are potentially comparible with psi-mi enzymatic reactions. Altogether 40,008 redundant protein interactions were extracted from KEGG xmls, from which 3044 phosphorylation, 627 dephosphorylation, 658 ubiquitination, 2 methylation and no glycosylation reactions were extracted. The first three categories were thus compared with our predictions using a hypergeometric p-value.

For computing Pearson correlation of protein abundance in pathways we choose KEGG’s subcategories consisting of at least five pathways, each of the pathways consists of at least ten protein interactions with a predicted interaction type.

### Network Motifs

In order to find network motifs, we exhaustively enumerated all patterns of three proteins interconnected with three PPIs, defined by a single interaction type per PPI. We compared the number of patterns found to those formed by 500 randomized PPI networks. Each random PPI network was created by permuting PPI edges of each interaction type separately, while maintaining the degree the proteins in the interaction-type network. The interaction type networks were then recombined into a random PPI as previously suggested by [18].

## Supporting Information

Table S1 **A list of detection methods over/under represented in different interaction types.**
(XLSX)

Table S2 **Detection methods used as features in the logistic regression.**
(XLSX)
